# Unveiling TRPV1 Spatio-Temporal Organization in Live Cell Membranes

**DOI:** 10.1371/journal.pone.0116900

**Published:** 2015-03-12

**Authors:** Barbara Storti, Carmine Di Rienzo, Francesco Cardarelli, Ranieri Bizzarri, Fabio Beltram

**Affiliations:** 1 NEST, Scuola Normale Superiore and Istituto Nanoscienze—CNR, Pisa, Italy; 2 Center for Nanotechnology Innovation @NEST, Istituto Italiano di Tecnologia, Pisa, Italy; University of Sydney, AUSTRALIA

## Abstract

Transient Receptor Potential Vanilloid 1 (TRPV1) is a non-selective cation channel that integrates several stimuli into nociception and neurogenic inflammation. Here we investigated the subtle TRPV1 interplay with candidate membrane partners in live cells by a combination of spatio-temporal fluctuation techniques and fluorescence resonance energy transfer (FRET) imaging. We show that TRPV1 is split into three populations with fairly different molecular properties: one binding to caveolin-1 and confined into caveolar structures, one actively guided by microtubules through selective binding, and one which diffuses freely and is not directly implicated in regulating receptor functionality. The emergence of caveolin-1 as a new interactor of TRPV1 evokes caveolar endocytosis as the main desensitization pathway of TRPV1 receptor, while microtubule binding agrees with previous data suggesting the receptor stabilization in functional form by these cytoskeletal components. Our results shed light on the hitherto unknown relationships between spatial organization and TRPV1 function in live-cell membranes.

## Introduction

The Transient Potential Vanilloid 1 or TRPV1 belongs to that fascinating class of polymodal membrane receptors that integrate several physical and molecular stimuli and convert them into efficient intracellular signaling [1]. More specifically, TRPV1 is a nonselective voltage-dependent, temperature-dependent, ligand-dependent cation channel with a preference for calcium ion[2]. Modulators of TRPV1 activity include: noxious heat [2], low pH [3], capsaicin and capsaicin analogues like resiniferatoxin (RTX) [4]. TRPV1 is a member of the transient receptor potential (TRP) channel family, which is typified by a predicted six transmembrane domain with intracellular N- and C-termini and a relatively-conserved pore domain [5,6]. TRPV1 is primarily expressed in sensory neurons, where it is involved in pain signaling [7]. Accordingly, this receptor is primarily studied to promote the design of drugs capable of controlling pain stress in humans [8].

Although many details of TRPV1 structure and functions have been revealed [9], the interaction network of TRPV1 with proteins related or embedded in the plasma membrane is still largely unknown. Very recently, we demonstrated the specific stabilization of functional membrane-bound TRPV1 by direct entanglement with microtubules [10]. While the role of activated TRPV1 in microtubule remodeling had been demonstrated earlier [11], our discovery showed for the first time a feedback cycle between nociception and cytoskeletal assembly/remodeling based upon long-term TRPV1 activation by agonists. Yet other-membrane related proteins might participate in regulating the nociception process. For instance, TRPV1 activation needs to be followed by the biochemical attenuation of nociceptive sensory neuron excitability, making TRPV1 partially or totally refractory to subsequent stimuli. From the analogy with other similar receptors, it has been suggested that long-term desensitization may involve TRPV1 withdrawal from the cell surface and its relocation inside the cell by some endocytotic mechanism. A recent study showed that TRPV1 activation promotes indeed receptor internalization by a clathrin-independent endocytotic mechanism [12]. The same authors speculated that caveolar endocytosis might be responsible for this process. This hypothesis was supported with the indirect observation that several TRPV1-related proteins (e.g. endothelial nitric oxide synthase, eNOS) are a well-characterized players of caveolin-mediated signaling pathways [13,14] both in cultured cells and *in vivo* [14,15]. Furthermore it was demonstrated that caveolin-1 interacts with and regulates exocytic trafficking of a member of Transient Receptor Potential the calcium channel TRPC1 (transient receptor potential channel 1) [16,17]. Caveolin-1 is also a major protein component of lipid rafts [18], i.e. membrane microdomains enriched in cholesterol and saturated lipids [19] known to modulate the activity of several receptors [20]. Notably a previous study highlighted the role of membrane cholesterol in preserving effective TRPV1 activity [21]. This still incomplete experimental picture raised the intriguing issue of a possible molecular partnership between TRPV1 and caveolin-1, and, if any, its relationship with the TRPV1 entanglement to microtubule.

In our previous work [10], we unveiled the TRPV1-microtubule interplay by combining molecular binding-sensitive Förster Resonance Energy Transfer (FRET) [22] confocal imaging with temporal Image Correlation Spectroscopy, the latter technique affording the average diffusion properties of membrane TRPV1 alone and when bound to microtubules. Experiments were carried out with cells that do not express endogenous TRPV1. The receptor was transiently expressed as fusion construct with a fluorescent-protein reporter that does not interfere with TRPV1 localization or activity [23–25].

In the present work, we expanded our approach targeting the spatial and temporal organization of membrane TRPV1 complexes with caveolin-1 and microtubules. This strategy was motivated by the need of a full and meaningful picture of receptor activity and regulation by these interactors. Although cell membrane was imaged selectively by Total Internal Reflection Fluorescence (TIRF) microscopy [26], our investigation relied on a novel acquisition method based on the extraction of the molecular mean square displacement directly from imaging data (*i*MSD) [27]. Thanks to this method we were able to probe the same physical quantities accessed by classical single-particle tracking but by using genetically-encoded fluorophores such as fluorescent proteins. We here show that *i*MSD in combination with FRET was able to yield a comprehensive picture of the spatio-temporal relationship of membrane TRPV1 with caveolae and microtubules. Our findings show that membrane TRPV1 is split, at least, in three pools with distinct functional roles, namely: 1) TRPV1-C, which is trapped in caveolae (binding to caveolin-1) and likely governs receptor long-term desensitization upon activation, 2) TRPV1-T, which is organized in large sub-micron membrane domains whose diffusion is modulated by microtubules and oversees the subtle feedback interaction between receptor and microtubules [10], and 3) TRPV1-I that diffuses on the membrane in a fast and isotropic fashion and might represent a reservoir of the receptor.

Our findings do unveil new and relevant properties of TRPV1 and may represent a paradigmatic model of how protein receptors, cytoskeleton and lipoproteic domains on the plasma membrane interplay to regulate cell functions and communication of cells with the external environment.

## Results and Discussion

### Spatiotemporal organization of TRPV1-caveolin (TRPV1-C) complex on the membrane

**Fig 1:**
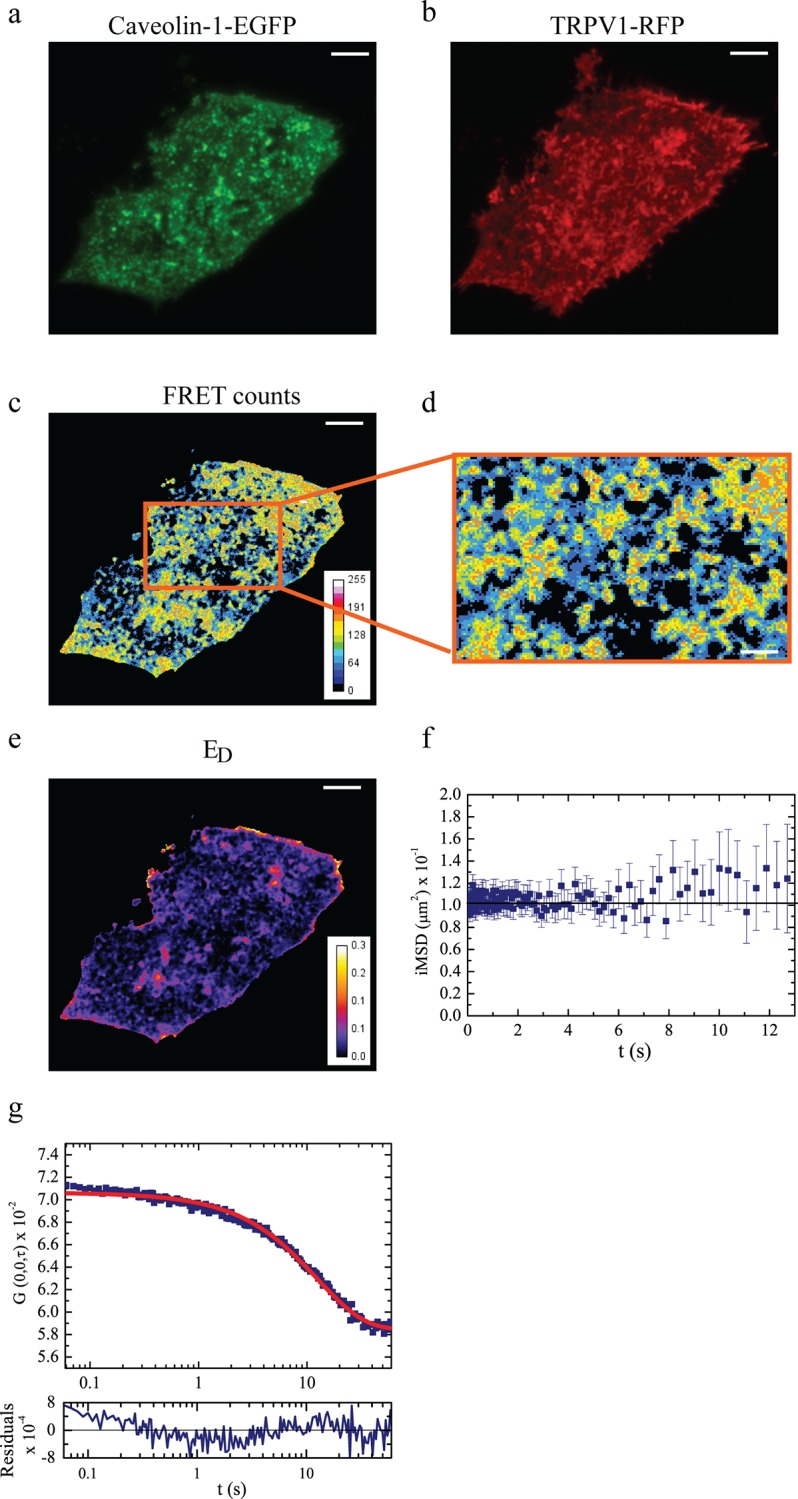
Steady-state and dynamic FRET analysis of TRPV1-Caveolin-1 interaction. Panel a-e refer to sensitized emission (steady state) FRET. (a) Donor (Caveolin-EGFP) emission image; (b) acceptor (TRPV1-RFP) emission image; (c) SE-FRET intensity image (scale bar: 5 μm); (d) zoom of a cellular region where FRET-*i*MSD analysis is later performed; (e) donor-normalized apparent FRET efficiency *E*_*D*._ Panels f-g refer to FRET-*i*MSD analysis. (f) *i*MSD vs. time plot of FRET signal for the Caveolin-1-EGFP/TRPV1-RFP pair in physiological condition. The constant *i*MSD clearly demonstrates that the complex is not diffusing in space, i.e. it is trapped within caveolin-positive structures on cellular membrane. (g) Temporal evolution of the binding component: plot of *G*(0,0,*τ*) vs. time shows the decrease of correlation function in time. This decrease of STICS function associated to a constant *i*MSD plot (f) confirms the presence of a binding interaction between TRPV1 and caveolin-1.

At first, we investigated whether TRPV1 and caveolin-1 directly interact with each other on the plasma membrane. To this end, we carried out Sensitized Emission FRET [28] (SE-FRET) experiments in CHO cells expressing TRPV1 and caveolin-1 genetically fused to spectrally-matched autofluorescent proteins. More specifically, caveolin-1 was fused with either one of two FRET donors: green-emitting EGFP [29] (Caveolin-EGFP) or yellow-emitting YFP (Caveolin-YFP). Red-emitting TagRFP was chosen as acceptor and was fused at the C-terminus to TRPV1 [30] (TRPV1-RFP). Imaging was performed in TIRF mode to address selectively the cell membrane. Experiments on doubly-labeled cells were preceded by the determination of the spectral bleed-through parameters (SBTs) in cells expressing only one construct at a time [28,31]. Nicely, in our set up we verified that both FRET pairs are associated with negligible SBT. For the donors we measured 1.7% and 3.3% for EGFP and YFP, respectively, while with the acceptor we obtained a SBT value of 3.3%. Following the classical Sensitized Emission FRET procedure [28], SBTs were applied to filter out the spurious fluorescence from the overall FRET emission as collected in doubly-labeled cells (see Methods section). Then the energy-transfer intensity was normalized by donor emission and scaled by donor-to-acceptor quantum yield ratio, to obtain an apparent FRET efficiency parameter (*E*_*D*_)which is independent from the excitation intensity [32] (S1 Text). Remarkably, a statistically significant SE-FRET (*p* < 10^−3^ with respect to a non-FRET system, see Materials and Methods and S1 Fig.) was obtained for both Caveolin-EGFP or Caveolin-YFP and TRPV1-RFP (Caveolin-EGFP: <*E*_*D*_ *>* = 4±2%, #17 cells, Caveolin-YFP: <*E*_*D*_ *>* = 30±6%, #10 cells, Fig. 1A-E). Although *E*_*D*_ is related also to the fraction donor engaged in the complex (S1 Text) and this figure could depend on the transfection efficiency of the caveolin construct, we note that the YFP-TagRFP couple was associated with a higher FRET because of the larger spectral overlap between the emission of the donor and the excitation of the acceptor. The presence of FRET was also confirmed by acceptor-photobleaching experiments (S2 Fig.). Here, we found *E*_*pb*_ *=* 11±3% (#6 cells) for Caveolin-EGFP / TRPV1-RFP, where *E*_*pb*_ stands for the apparent FRET percentage calculated as in [33]. We note that the negative FRET control gave *E*_*pb*_ *=* -0.006±0.004% (#3 cells). These findings unequivocally demonstrate the existence of a complex between TRPV1 and Caveolin-1 on the cell membrane, which will be hereafter referred to as TRPV1-C.

As anticipated in the introductory section, diffusion and binding features of target fluorescent molecules can be effectively investigated by spatiotemporal image correlation spectroscopy according to a novel approach denoted as *i*MSD [27]. A comprehensive description of the *i*MSD is reported in S2 Text. In short, *i*MSD experimentally requires a temporal stack of images of the sample. On this stack, the image spatiotemporal autocorrelation function *G*(*ρ*,*τ*) is calculated at variable spatial (*ρ*) and temporal (*τ*) increments, like in STICS technique [34,35]. The minimum step of these increments is unequivocally determined by the pixel size dimensions and acquisition time between each image. *G*(*ρ*,*τ*) is usually represented by a Gaussian distribution along the spatial dimension *ρ*, and the temporal pattern of its variance is the actual *i*MSD trace. The *i*MSD approach readily gives access to four relevant parameters: 1) the diffusion coefficient of the observed species (*D*), 2) an indicator (*α*) measuring the deviation of the diffusion process from brownian behavior, 3) an estimate of the molecular dimension of diffusing species (*d*), and 4) the characteristic time of binding (*τ*_*b*_) if a reversible complex is under observation. FRET can be combined to *i*MSD (FRET-*i*MSD) to return selectively the diffusional/binding parameters of the molecular complex for which the energy transfer between fluorophores is observed. In this context, the EGFP / TagRFP pair is particularly effective, as the minimal SBTs affect only negligibly the FRET-*i*MSD autocorrelation traces (S3 Fig.) and only the features relevant to the complex are afforded.

Remarkably, *i*MSD of TRPV1-C was found to be rather flat indicating no appreciable spatial movement of the complex (Fig. 1F). Yet, the purely temporal autocorrelation *G*(0, *τ*) dissipated with time, indicating that TRPV1-C is labile from the temporal point of view (Fig. 1G). Taken together, these results demonstrate that TRPV1 and caveolin-1 do not co-diffuse significantly as a complex, although TRPV1-C undergoes dissociation as expected for a reversible binding process. By fitting *G*(0, *τ*) to the binding model represented (Equation M in S2 Text), we obtained τ_*b*_ = 16.4±4.0 s (# 8 cells, Table 1). Furthermore, calculation of *d* (Equation N in S2 Text) from *i*MSD and the spatial resolution of the imaging apparatus in the focal plane indicated that TRPV1-C is localized to membrane patches with an apparent radius of about 150–200 nm (see also Fig. 1D, zoom).

**Table 1:** Parameters derived from iMSD analysis.

**TRPV1 pool**	***D* (μm^2^/s)**	***α***	***d* (nm)**	**τ_*b*_ (s)**	**#cells**	**A (%)**	
**TRPV1-C**	∼ 0	-	360±40	16.4±4.0	22	68±21	**Basal**
	*	*	*	*	8	69±14	**+NDZ**
	*	*	*	2.9±2.1	10	88±08	**+SMase**
**TRPV1-T**	(9.0±2.6) ∙10^−3^	1.5±0.3	860±300	»60 s	22	19±16	**Basal**
	-	-	-	-	-	0	**+NDZ**
	-	-	-	-	-	0	**+SMase**
**TRPV1-I**	0.22±0.02	1	-	-	22	13±09	**Basal**
	0.99±0.18	*	-	-	8	31±15	**+NDZ**
	0.10±0.04	*	-	-	10	12±07	**+SMase**

The rows indicate the different dynamic populations of TRPV1. For each population we describe three conditions: basal, upon nocodazole (+NDZ) or sphingomyelinase (+SMase) administration. For the columns, we report dynamic parameters from left to right: Diffusion coefficient (D), anomalous diffusion exponent (*α*), diameter of diffusing species (d), binding time (τ_*b*_) and amplitude of populations (A). The values are expressed as Mean±Standard Error.

* Values keeping equal at basal condition (obtained by STICS-FRET analysis) during the fitting.

- Not Determinate.

### Spatiotemporal organization of TRPV1-microtubule (TRPV1-T) complex on the membrane

**Fig 2:**
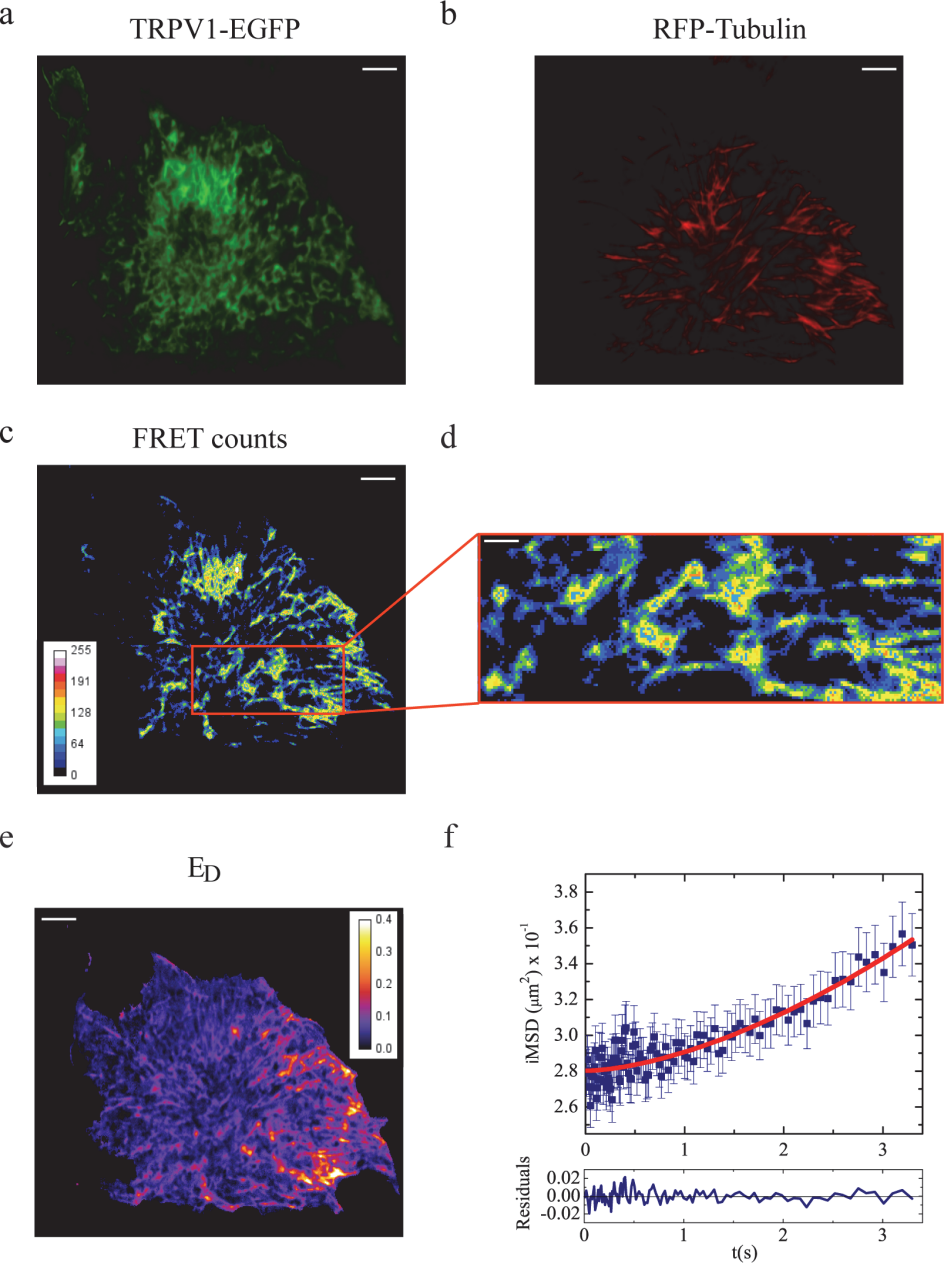
Steady-state and dynamic FRET analysis of TRPV1-microtubules interaction. Panels a-e refer to sensitized emission (steady state) FRET. (a) Donor (TRPV1-EGFP) emission image; (b) acceptor (tubulin-RFP) emission image; (c) SE-FRET intensity image (scale bar: 5 μm); (d) zoom of a cellular region where FRET-*i*MSD analysis is later performed (scale bar: 1 μm); (e) donor-normalized apparent FRET efficiency *E*_*D*._ Panel f refers to FRET-*i*MSD analysis. (f) *i*MSD vs. time plot for FRET signal between TRPV1-EGFP and RFP-tubulin in physiological condition.

As expected from our previous results [10], in static condition TRPV1 and *α*-tubulin gave a well-detectable SE-FRET signal (Fig. 2A-E), thereby demonstrating the existence of a complex (hereafter denoted as TRPV1-T). This interaction was analyzed from the spatiotemporal point of view by the same FRET-*i*MSD modality adopted for TRPV1-C using EGFP-labeled TRPV1 and TagRFP-labeled *α*-tubulin. Remarkably, in this case, *i*MSD of TRPV1-T was found to be associated with hyperbolic growth with time (Fig. 2F). This "super-diffusive" behavior is attributable to guided/active mobility [36], and was characterized by *D* = (9.0±2.6) ∙ 10^−3^ μm^2^/*s* ^α^ and *α* = 1.5±0.3 (Table 1). No traces of a binding-related dissipation of autocorrelation amplitude with time was detected, indicating that the characteristic time of binding should be much longer of the overall acquisition time of the measurements (60 s, Table 1). Interestingly, we found *d* ≈ 400 nm as the radius of the super-diffusive TRPV1-T (Table 1). This finding suggests that the interaction between the two components occurs across a large sub-micron domain of cell membrane. Consistently, a highly patterned signal is visible also in the static FRET image that is composed by several spots with 500–1000 nm size (Fig. 2D, zoom).

### Spatiotemporal organization of global TRPV1 pool on the membrane

**Fig 3:**
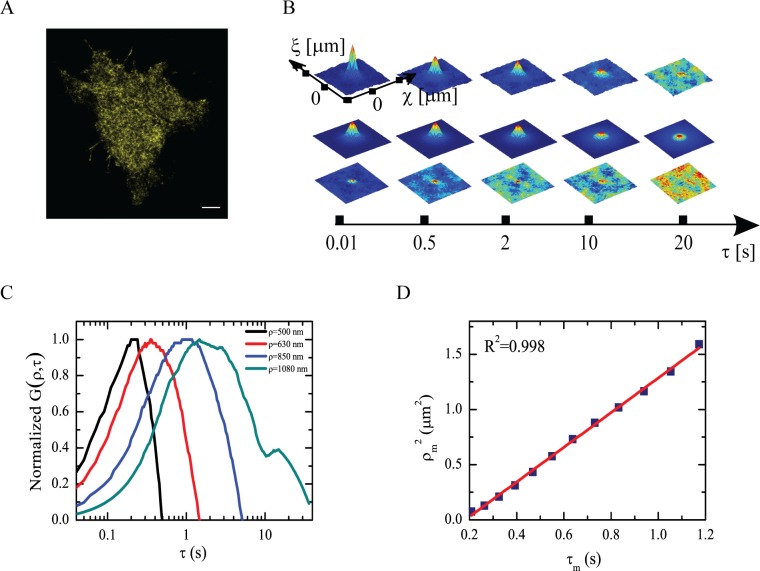
Disclosing of an isotropic TRPV1 component by *i*MSD analysis on cell transfected with TRPV1-YFP alone. (a) TIRF microscopy image of a CHO cell expressing TRPV1-YFP. (b) Temporal evolution of the correlation function with the corresponding Gaussian fit and residues. The fitting is a sum of two components that account for binding and superdiffusion regimes. The residuals at short timescale are non-zero and they can be attributed to diffusion of TRPV1 molecule not directly involved in tubulin or caveolin interactions. (c) Average normalized correlation functions of TRPV1 for distances between about 500 nm e 1 μm. The data are smoothed. The position of the maximum of the curves moves at increasing of distance along the time. The shift in the maximum indicates spatial spreading of observed molecules. (d) Plot of position of each maximum identified by $\rho_{m}^{2}$ and τ_*m*._ The linear trend (R^2^ = 0.998) indicates free diffusion at this spatial scale of the TRPV1-I pool.

The observed presence of two distinguishable pools of TRPV1, (TRPV1-C and TRPV1-T) prompted us to investigate whether they make up for the total amount of TRPV1 in the cell membrane. To do this, we carried out *i*MSD measurements on TRPV1-YFP without labeling any other cellular component (Fig. 3A). More specifically, we fitted the autocorrelation functions with a sum of components accounting for a binding regime (TRPV1-C) and a super-diffusive regime (TRPV1-T) (Fig. 3B). Notably, inspection of fitting residuals highlighted that the global pool of TRPV1 is not composed solely by these two components at short times (< 2 s; Fig. 3B). To highlight the nature of this fast component, we studied the shape of the spatiotemporal correlation function *G*(*ρ*,*τ*). Fig. 3C shows plots of *G*(*ρ*,*τ*) *vs*. *τ* for 500 nm < *ρ* < 1 μm. Notably, each curve is characterized by a maximum that occurs at longer times at increasing *ρ* values. We identify this maximum by the coordinates (*ρ*_*m*_,*τ*_*m*_). In S2 Text we demonstrate that the $\rho_{m}^{2}$ *vs*. *τ*_*m*_ plot reflects only the diffusive behavior of TRPV1 with no contribution by its reversible binding interactions. Significantly, for short times (<2 s) the $\rho_{m}^{2}$ *vs*. *τ*_*m*_ plot was found to display an almost linear behavior (Fig. 3D) thus indicating a prevalent isotropic diffusion regime with *D* = (2.2±0.2) ∙10^−1^ μm^2^/s. We should note that in this time window the guided diffusion by microtubules is spatially negligible since the square spatial displacement of TRPV1-T is less than 0.03 μm^2^ and does not contribute to the observed *i*MSD. We must therefore conclude that the TRPV1 pool entails an additional fast, isotropically-moving fraction (hereafter denoted as TRPV1-I) beside the spatially-confined TRPV1-C and the slow-moving TRPV1-T guided by microtubules. We identify TRPV1-I with the dimeric fast-moving receptor pool observed in our previous work [10].

**Fig 4:**
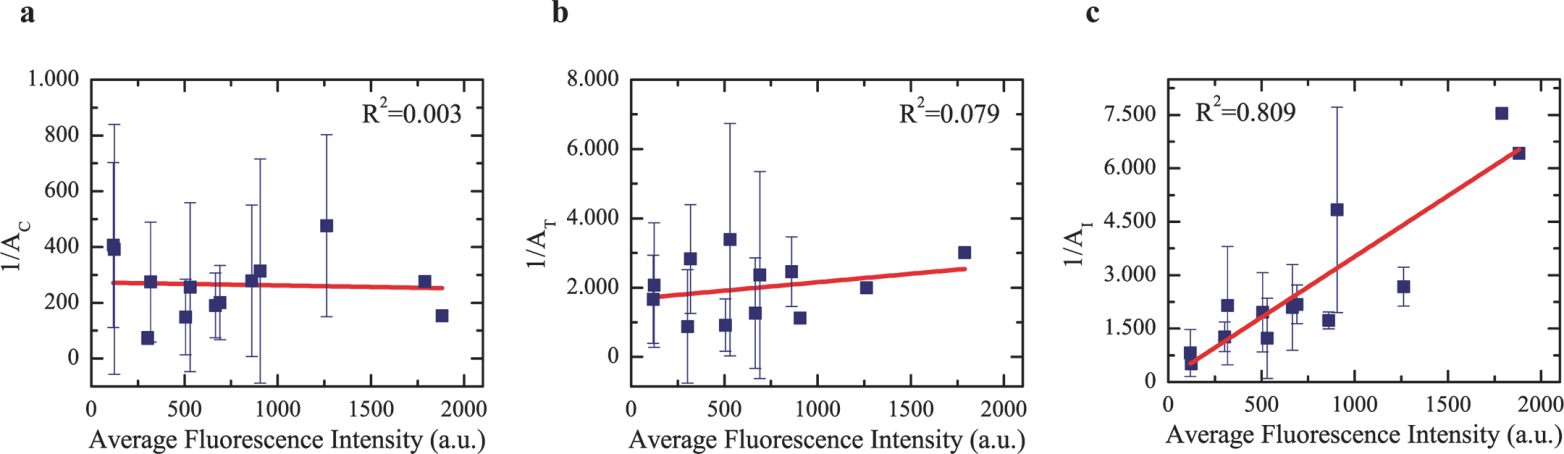
Dependence of TRPV1 components upon cellular expression level in living cells expressing TRPV1-YFP. The graphs show the inverse of relative amplitudes of the three components of TRPV1 *vs*. average fluorescence intensity; the latter quantity is proportional to protein expression level in living cells. This analysis reveals the TRPV1-C and TRPV1-T components to be negligibly affected by the expression level (a and b respectively) while there is a positive dependence of the average intensity for the isostropic component TRPV1-I (c, with R^2^ = 0.809).

Next, we set out to determine the relative contributions of the three TRPV1 pools to its spatiotemporal dynamics. To do this, we included the isotropic TRPV1-I pool to the sum accounting for the autocorrelation function *G*(*ρ*,*τ*). By fitting the actual data (S4 Fig.), we calculated the relative autocorrelation amplitudes *A*_*C*_, *A*_*T*_, and *A*_*I*_ relevant to TRPV1-C, TRPV1-T, and TRPV1-I, respectively. Although these values cannot be straightforwardly translated into mole fractions owing to the wide range of factors that contribute to determine the correlation amplitude [37], their changes with the average fluorescence intensity collected on the membrane afford a semi-quantitative picture on how the three TRPV1 pools are populated at different global receptor concentration (S2 Text). Interestingly, we found out that *A*_*C*_ *A*_*T*_ have a negligible dependence from the average fluorescence intensity of TRPV1-YFP (i.e. from the expression level of the receptor) (Fig. 4A-B), as witnessed by a linear correlation coefficient *r* ^*2*^ < 0.1. Conversely, *A*_*I*_ is significantly correlated (*r* ^*2*^ > 0.8) with TRPV1 membrane concentration (Fig. 4C). These findings suggest that the membrane concentrations of TRPV1-C and TRPV1-T are under strict biochemical control and cannot be changed by protein over-expression, as expected for functional cellular structures. Instead, concentration of TRPV1-I seems dependent on the protein expression level suggesting a different role for this component, possibly as TRPV1 “reservoir”. Nicely, upon nocodazole treatment and disassembling of microtubules, the super-diffusion population disappeared and the correlation function was fitted by just two components: TRPV1-C and TRPV1-I (S5 Fig.). It is worth comparing the average correlation amplitudes (as calculated in a narrow membrane concentration range of TRPV1) at basal state and in presence of nocodazole (Table 1). Remarkably, *A*_*C*_ was unaffected by nocodazole while *A*_*I*_ appeared to increase. As a consequence we can state that the TRPV1 previously occupied in the interaction with tubulin merged with the isotropic pool after microtubules disassembling. Additionally, nocodazole was found to raise significantly the diffusion coefficient of TRPV1-I on the membrane (Table 1), an effect already demonstrated for the receptor at global level in our previous work [10]. Thus, microtubule disassembling seems to lead to a more fluid membrane environment for TRPV1-I diffusion.

Eventually, we tested the effect of sphingomyelinase on TRPV1 components, to assess the role of sphingomyelin-enriched membrane rafts, including caveolae, on receptor dynamics. As expected, sphingomyelinase hampered the formation of TRPV1-C, as witnessed by the significant 5-fold decrease of τ_*b*_ (Table 1). Surprisingly, however, sphingomyelinase triggered the disappearance of TRPV1-T component. This is witnessed by the successful fit of *G*(*ρ*,*τ*) by the binding and isotropic components alone (S6 Fig.), and by the reduction of $\sigma_{0}^{2}$ to values comparable with the instrumental waist allowing us to exclude the presence of the large aggregates observed in the interaction with microtubules (Table 1). We should also note that the amount of TRPV1-I was not significantly affected, although its diffusion coefficient was more than halved. These findings suggest that removal of sphingomyelin has a strong specific effect on large membrane patches containing TRPV1-T and a more systemic influence on membrane fluidity.

### Caveolin-mediated TRPV1 internalization upon agonist activation

To complete the picture of TRPV1 interaction with caveolin-1, we set out to demonstrate whether clathrin-free receptor internalization upon activation is indeed mediated by caveolin-1 and follows the caveolar pathway. For this goal, we compared quantitatively the colocalization degree between caveolin-1-EGFP and TRPV1-RFP before and after activation in the internal part of the cell. Imaging was carried out by conventional confocal microscopy.

**Fig 5:**
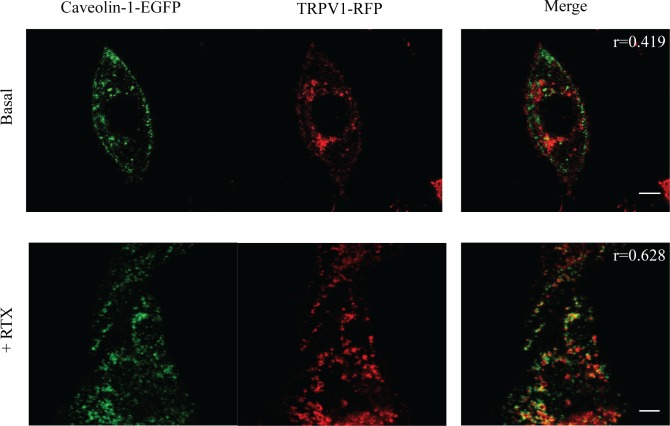
Colocalization experiments between caveolin-1-EGFP and TRPV1-RFP inside cells. Colocalization between caveolin-1-EFGP and TRPV1-RFP in CHO cells before and after RTX administration. Left panel, Caveolin-1-EGFP emission image; middle panel, TRPV1-RFP emission image; right panels, composite image between Caveolin-1-EGFP and TRPV1-RFP images; the values r in the pictures indicate the Pearson coefficient of images. Scale bar for basal state image: 7.5 μm; scale bar for activated state image: 5 μm.

At basal state (Fig. 5), caveolin-1-EGFP and TRPV1-RFP show almost negative colocalization in the internal regions of CHO cells as witnessed by the average Pearson coefficient <r> = 0.470.05 (#5 cells) We should remind that an accepted criterium relates r > 0.5 to positive colocalization [38]. Yet, a few minutes after administration of the agonist RTX to the same cells, caveolin-1-EGFP was found to colocalize, although not completely, with activated TRPV1-RFP (<r> = 0.700.09, #10 cells) in intracellular regions that should be identified as caveolar vesicles and caveosoma (Fig. 5). The statistical difference between basal and activated states was confirmed also by Student’s *t*-test between the two relevant datasets, for which *p* < 10^−4^. Also in light of previous data on endocytic internalization of TRPV1 [12], we may interpret the binding of TRPV1 to caveolin-1 as a biological means to trigger receptor de-sensitization upon prolonged activation via caveolar endocytosis. This behavior is shared also by other membrane receptors [39,40]. This conclusion seems supported also by the reversible TRPV1 binding to caveolin-1 on the membrane with a characteristic binding time of several seconds (Table 1).

### Role of membrane structure and working model of TRPV1-protein interaction

Several dynamic and spatial features of TRPV1 pools on membrane are worthy of note. First, TRPV1-T appears to move within the membrane in rather large patches of size around 800–900 nm (Table 1 and Fig. 2). Note that the cytoplasmic tail of a single TRPV1 chain is known to possess a binding stretch to the plus end of microtubules [41]. Thus, it appears that TRPV1-T consists of the supramolecular arrangement of several TRPV1 receptors, each one linked to a microtubule chain (as assessed by FRET). This system is characterized by a motion actively driven by microtubule chains, as witnessed by the absence of "guided" diffusion when microtubules are disassembled. Moreover sphingomyelin is a necessary constituent of the membrane regions including TRPV1, as demonstrated by the disappearance of the TRPV1-T pools upon sphingomyelin digestion by sphingomyelinase. These findings suggest the raft nature of the membrane patches where TRPV1-T is located, in agreement with a previous study that highlighted the correlation between cholesterol presence in the membrane and effective TRPV1 activity [42]. Although more experimental data are probably necessary to fully clarify the structural features of TRPV1-T, we can sketch a first model in which many TRPV1 molecules move, in a coordinated way, either along microtubule chains that reside underneath large membrane patches, or jointly with microtubule tethers.

Second, one may question whether the regions containing caveolin-bound TRPV1 are subsets of these large TRPV1-T patches, on account of the recognized interplay between caveolae and microtubules [43,44]. Our findings suggest that TRPV1-C and TRPV1-T are *spatially decoupled*: in fact, the *i*MSD trace at time zero of TRPV1-C did not display large patch-like arrangements as in the case of TRPV1-T, a feature that would be expected if the two TRPV1 pools were accommodated in the same membrane location. Additionally, microtubule disassembly did not affect the amplitude of TRPV1-C correlation, while it was found to increase the amplitude of the freely-diffusing pool TRPV1-I. Although correlation amplitudes should not be correlated straightforwardly to mole fractions [37], this result is a further suggestion that TRPV1-C and TRPV1-T do not spatially overlap. This spatial and functional decoupling is particularly relevant and deserves additional comments. Based on experimental evidences by us and others, Goswami recently suggested a model in which TRPV1 assembles firstly as a dimer, then tetramerizes, and is eventually sensitized towards agonist activation by phosphorylation [45]. In this model, microtubules promote tetramerization but are detached from TRPV1 upon phosphorylation. Although experimental evidence is incomplete, it is tempting to identify the sensitized receptor with the pool binding to caveolin-1. This mechanism would nicely link sensitization upon phosphorylation and de-sensitization upon internalization, thereby accounting for the fine regulation of receptor activity required by its biological role.

## Conclusions

In conclusion, for the first time here we unveiled the spatio-temporal regulation of TRPV1 dynamics within the plasma membrane. Our observations showed that there are three population of TRPV1 with rather different properties: 1) TRPV1-C capable of binding with caveolin-1 and internalizing after activation, 2) TRPV1-T interacting with membrane tubulin and organized into sub-micrometric plasma-membrane domains; 3) TRPV1-I a freely fast-diffusing fraction. Only TRPV1-I shows a non-negligible correlation between its relative abundance and the expression level of the receptor, suggesting its role as TRPV1 reservoir. The discovery of caveolin-1 as a new interactor of TRPV1 and the observation of positive colocalization of caveolin-1 and TRPV1 after receptor activation suggests that caveolar endocytosis is an important desensitization pathway of TRPV1 receptor upon activation. Other desensitization pathways cannot be ruled out, however, on account of the observed non-complete colocalization of activated TRPV1 and caveolin-1 inside cells. Furthermore we provided novel information on the peculiar interplay between the spatial organization of TRPV1 and the microtubular component of cell cytoskeleton. The spatial and functional decoupling between TRPV1-T and TRPV1-C may be interpreted in terms of a recently-proposed model, where TRPV1 is sensitized to agonist binding by phosphorylation and microtubule detachment. We believe that the present results can provide useful insight onto the hitherto unknown spatial organization of TRPV1 in live cell membranes and their relation with receptor function.

## Methods

### Cell Cultures, Constructs and Transfection

CHO K1 cells were provided by American Type Culture Collection (CCL-61 ATCC) and grown in Dulbecco’s modified Eagle medium F-12 nutrient mix (D-MEM/F-12, Invitrogen, Carlsbad, CA) supplemented with 10% fetal bovine serum, 100 U/ml penicillin, and 100 mg/ml streptomycin (Invitrogen). For live imaging, 12×10^4^ cells were plated 24 hours before transfection onto a 35-mm glass-bottom dish (WillCo-dish GWSt-3522). Cells were imaged 24 hours after transfection. Transfections of all constructs were carried out using Lipofectamine reagent (Invitrogen) according to the manufacturer’s instructions. In all experiments, cells were maintained at 37°C in a 5% CO_2_ atmosphere. The observed channel localization within CHO cells transiently transfected with fluorophore-fused proteins reproduces the channel localization in endogenous system and the fused protein does not alter channel functionality. [46–49] Activation of TRPV1 in colocalization experiments was assessed following stimulation with 20 nM of RTX (resiniferatoxin, Sigma Aldrich) of cells transfected with Caveolin-1-EGFP and TRPV1-TagRFP. Acquisitions were performed after 5 minutes of administration and cells were kept in D-MEM/F-12 nutrient mix during the measurements. In microtubule-depolimerization experiments cells were incubated in fresh medium with 3mg/ml of nocodazole (Sigma Aldrich) for 10 minutes. In order to disrupt sphingomyelin on the plasma cellular membrane we used 1 U/ml Sphingomyelinase from *Bacillus cereus* (Sigma Aldrich) for 30 minutes. Caveolin-1-EGFP and Caveolin-1-YFP were kind gifts by Prof. Pelkmans [50]. All details about the TRPV1-YFP construct are described in Ref [10]. The TRPV1-EGFP construct was generated by site-direct mutagenesis of p-TRPV1-E^0^GFP (described in ref. [10]) using the following primer: S65T, 5’-GTGACCACCCTGACCTACGGCGTGCAGTGCTTC-3’. The TRPV1-RFP construct was obtained by sub-cloning TRPV1 sequence from TRPV1-YFP construct in pTagRFP-N (Evrogen, catalog no. FP142) cut by HindIII and SmaI. RFP-tubulin construct is provided from Evrogen (catalog no FP145).

### Total Internal Reflection Microscopy (TIRF-M)

Imaging in Total Internal Reflection mode (TIRF-M) was carried out by a Leica AF6000 fluorescence microscope equipped with TIRF-M condenser and 100x oil-immersion objective (NA 1.47). We adjusted the penetration depth of the evanescent wave to less than 100 nm. Fluorescence was recorded by a cooled EM-CCD (Hamamatsu C1900–13). The microscope was equipped with laser lines for excitation of EGFP and YFP (488 nm) and TagRFP (561 nm). Acquisition settings were as in the following:

- EGFP and YFP: excitation was set to 488 nm, the emission was collected between 520 and 550 nm, and a dichroic filter at 502 nm separated out excitation from emission;
- Tag-RFP: excitation was set at 561 nm, emission was collected by a superposition of 600/40 641/75 and LP650 filters (yielding light at 604–620 and 650–679 nm), and a dichroic filter at 570 nm separated out excitation from emission;
- FRET (SE-FRET and FRET-*i*MSD): excitation was set to 488 nm, the emission was collected as for TagRFP, and a dichroic filter at 502 nm separated out excitation from emission;

### Sensitized emission FRET

Sensitized emission FRET measurements were carried out in TIRF-M mode by sequentially collecting (total time, 3 seconds) three images: 1) Donor image *F*_*D*_ (acquisition setting EGFP or YFP), 2) Acceptor image *F*_*A*_ (acquisition setting TagRFP), and 3) FRET image *F*_*FRET*_ (acquisition setting FRET). Radiation intensity due solely to energy transfer (*F*_*SE*_) was calculated for each pixel by:

$$F_{SE}=F_{FRET}-a\cdot F_{D}-b\cdot F_{A}$$ (1)

where *a* and *b* are parameters accounting for average bleed-through of donor and acceptor and were determined on cells expressing either the donor or the acceptor alone [28]. More precisely:

$$a=\frac{{\left(F_{D}\right)}_{A}}{F_{D}}\;\text{and}\;b=\frac{{\left(F_{A}\right)}_{D}}{F_{A}}$$ (2)

where (F_D_)_A_ and (F_A_)_D_ represent the donor and acceptor emissions in the acceptor (FRET) collection channel upon excitation at the donor wavelength, respectively. For EGFP and YFP we found *a* = 0.017 and *a* = 0.033, respectively. For RFP, *b* = 0.033.

Finally, for each pixel we divided the actual energy transfer intensity *F*_*SE*_ by the intensity collected in the donor channel *F*_*D*_, and multiplied this ratio by the ratio between the quantum yields of EGFP or YFP and TagRFP, to yield a normalized value *E*_*D*_ that is independent of excitation intensities (S1 Text).

$${\bar{F}}_{SE}=\frac{F_{SE}}{F_{D}}\cdot \frac{\Phi_{A}}{\Phi_{D}}$$ (3)

SE-FRET calculations were carried out in batch processing mode by an homemade plugin of the ImageJ software, as described in ref. [10]. Prior to SE-FRET calculations, donor and acceptor images were intensity-thresholded by the Li’s cross-entropy method [51] to remove low-intensity pixels that would generate erroneous SE-FRET and *E*_*D*_ values. Positive FRET data were compared with negative control measurements involving EGFP or YFP together with TRPV1-RFP. The statistical significance of positive FRET was ascertained by an unpaired, two-tailed, Student’s *t*-test yielding *p* = 2.10^−6^ (Caveolin-EGFP) and *p* = 4.10^−4^ (Caveolin-YFP).

### Acceptor photobleaching FRET

Acceptor photobleaching FRET measurements were carried out in TIRF-M mode by sequentially collecting donor image *F*_*D*_ (acquisition setting EGFP or YFP) and acceptor image *F*_*A*_ (acquisition setting TagRFP), before and after acceptor photobleaching by strong illumination at 561 nm (wide-field modality to ensure extended bleaching cell-wide). The FRET percentage was computed following Stepensky [33].

### iMSD and FRET-iMSD

For *i*MSD (acquisition setting: YFP) and FRET-*i*MSD (acquisition setting: FRET) measurements, TIRF time series were acquired at 10 ms time resolution by using the overlapping mode of EM-CCD camera. Typical time series lasted at least 6000 frames (∼60 seconds). *i*MSD analysis was performed by an homemade MatLab library as previously shown [27]. The contribution to the STICS function of an immobile fraction of particles was removed by proper filtering [34]. Additional possible artifacts due to average image intensity temporal drift, were removed by subtracting the spatial-averaged image, as described in Ref [52].

The mathematical description of *i*MSD method are reported in S2 Text. For data analysis of autocorrelation function deriving from FRET signal we applied Equation P in S2 Text that account for both diffusion and binding of the complex. When diffusion was into play, $\sigma_{r}{}^{2}\left(\tau \right)$(*i*MSD trace) was fitted to Equation H in S2 Text to recover the diffusion coefficient *D* and the diffusion parameter *α*. The *i*MSD value at time zero ($\sigma_{0}^{2}$) was converted into the dimension of the diffusing species *d* by Equation I in S2 Text, or Equation N in S2 Text for pure binding, after determination of the resolution of the imaging apparatus in the focal plane σ_*xy*_. The negligible influence of donor or acceptor spectral bleed-throughs on the autocorrelation of FRET signal was tested on cells expressing TRPV1-EGFP or TRPV1-TagRFP alone.

The autocorrelation signal of TRPV1-YFP alone was fitted by Equation O in S2 Text assuming three components: TRPV1-C, TRPV1-T, and TRPV1-I. In this case Equation O in S2 Text becomes:

$$G\left(\rho ,\tau \right)=A_{C}G_{C}\left(\rho ,\tau \right)+A_{T}G_{T}\left(\rho ,\tau \right)+A_{I}G_{I}\left(\rho ,\tau \right)$$ (4)

where *G*_*C*_(*ρ*,*τ*) is the correlation function of a binding species [27] (Equation M in S2 Text) and *G*_*T*_(*ρ*,*τ*) are diffusive component as defined in Equation G in S2 Text. For each component, the characteristic parameters (*D*, τ_*b*_, σ_0_, *α*) were fixed to the average values of Table 1 and we subsequently fitted the autocorrelation function to equation (4), thereby obtaining *A*_*C*_, *A*_*T*_ and *A*_*I*_.

### Colocalization studies

Colocalization studies were carried out in confocal imaging mode by a Leica TCS SP5 SMD confocal microscope (Leica Microsystems, Mannheim, Germany) using a 40x (NA 1.25) Plan Apo oil immersion objective while setting the confocal pinhole at 1 Airy unit. The microscope was interfaced with sources for the excitation of EGFP (488 nm, Ar laser line) and TagRFP (561 nm, HeNe laser line). The images were collected using low excitation power at the sample (10–20 μW) Concomitant fluorescence emissions in EGFP (490–525 nm) and TagRFP (610–660 nm) channels was acquired in spectral mode by the collection system of the microscope based on photomultipliers and Acousto-Optic Beam Splitters as dichroic filters. Image format was 512 x 512 pixels and line scanning speed was set to 400 Hz. 8–32 line averages were collected in all cases.

Colocalization between EGFP and TagRFP images was analyzed by the Image Correlation Analysis plugin of the ImageJ software (National Institutes of Health, Bethesda, MD).

## Supporting Information

S1 Fig FRET negative control. CHO cells were co-transfected with EGFP and TRPV1-RFP and SE-FRET analysis was carried out. Left panel: Donor (EGFP); middle panel: acceptor (TRPV1-RFP); right panel SE-FRET intensity. The FRET image show the absence of significant FRET signal. Scale bar: 5 μm.
(TIF)

S2 Fig FRET by acceptor photobleaching. A CHO cell expressing Caveolin-1-EGFP (Donor) and TRPV1-RFP (acceptor) was imaged before and after extensive acceptor photobleaching by strong illumination at 561 nm in epi-fluroescence modality.
(TIF)

S3 Fig *i*MSD of TRPV1-RFP in FRET mode. (a) TRPV1-RFP image obtained by exciting at 561 nm and acquiring between 604–620 and 650–679 nm (b) Image of the same cell acquired by exciting at 488 nm and acquiring between 604–620 and 650–679 nm. (c) Correlation function temporal evolution of image stack acquired in FRET mode (excitation at 488 nm and acquisition between 604–620 and 650–679 nm).
(TIF)

S4 Fig Correlation function temporal evolution with the corresponding Gaussian fit and residues including the global fit of TRPV1-I with TRPV1-C and TRPV1-T. The upper row represent the correlation function, the middle the fitting and the lower row residuals. The fitting is a sum of three components that account for isotropically diffusion, binding and superdiffusion regimes.
(TIF)

S5 FigTRPV1 populations organization in the presence of nocodazole.(a) Correlation function temporal evolution with the corresponding Gaussian fit and residues. The fitting is a sum of two components that account for binding and isotropic diffusion regimes. The superdiffusive TRPV1-T was not considered for the absence of intact microtubule. The residuals show that this model well described the situation in presence of nocodazole. (b) For the identification of the isotropic specie representative average correlation functions of TRPV1 for distances between about 500 nm e 1.5 μm were analyzed. The position of the maximum of the curves moves at increasing of distance along the time. The data are smoothed. The shift in the maximum clearly indicate spatial spreading of observed molecules. (c) Plot of position of each maximum identified by $\rho_{m}^{2}$ and τ_*m*._ The linear trend (R^2^ = 0.995) clearly indicates the free diffusion in this spatial scale of TRPV1-I pool with a different diffusion coefficient in respect to basal condition (*D* = 0.99±0.18 μm^2^/s).(TIF)

S6 Fig *i*MSD analysis of CHO cells transfected with TRPV1-YFP after sphingomyelinase treatment. (a) Temporal evolution of the correlation function with the corresponding Gaussian fit and residues. The fitting is a sum of two components that account for binding and free diffusion regimes. (b) Temporal evolution of the components: plot of *G*(0,0,*τ*) vs. time.
(TIF)

S1 Text Donor-normalized sensitized emission FRET. (PDF)

S2 Text *i*MSD approach to spatiotemporal image correlation spectroscopy (STICS). (PDF)
