# Supplementary figures and images for: Unveiling TRPV1 Spatio-Temporal Organization in Live Cell Membranes
Source: PLoS One. 2015 Mar 12;10(3):e0116900. doi: 10.1371/journal.pone.0116900 (PMC4357434; doi:10.1371/journal.pone.0116900)

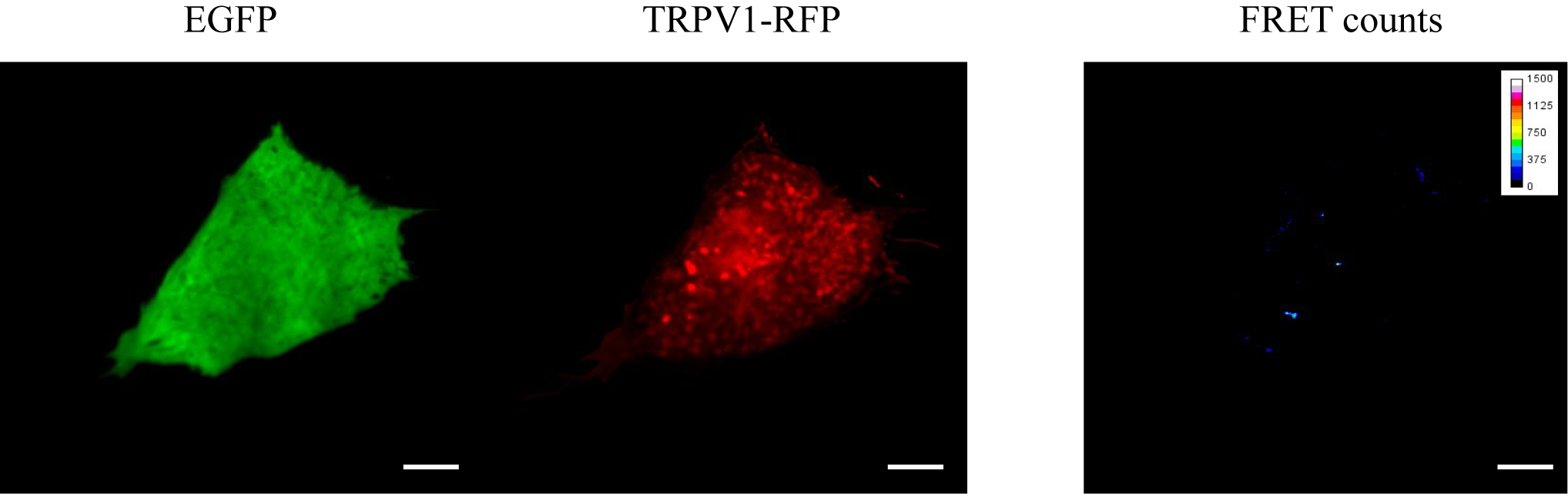

Supplement: S1 Fig — CHO cells were co-transfected with EGFP and TRPV1-RFP and SE-FRET analysis was carried out. Left panel: Donor (EGFP); middle panel: acceptor (TRPV1-RFP); right panel SE-FRET intensity. The FRET image show the absence of significant FRET signal. Scale bar: 5 μm. (TIF) [file pone.0116900.s001.tif]

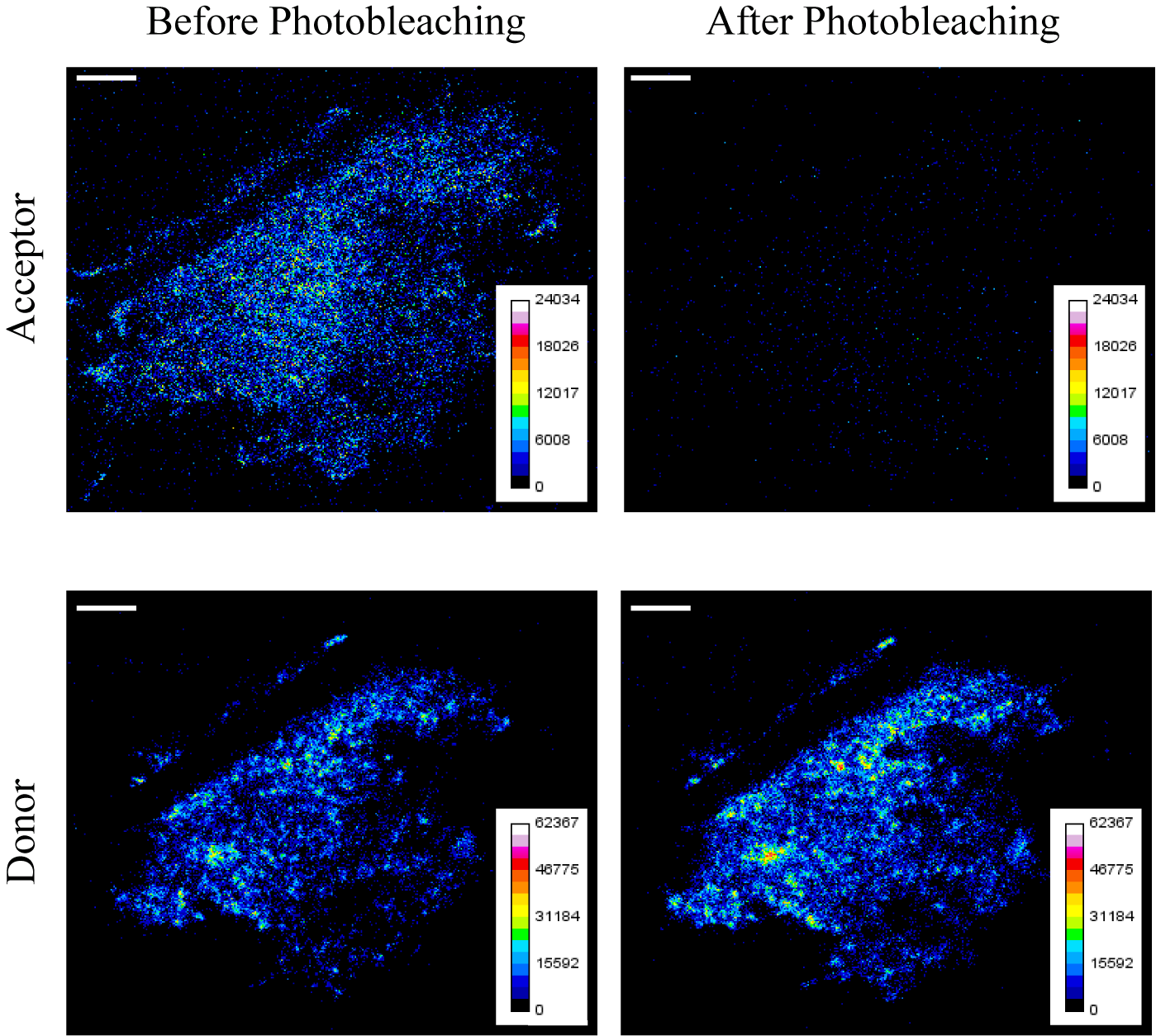

Supplement: S2 Fig — A CHO cell expressing Caveolin-1-EGFP (Donor) and TRPV1-RFP (acceptor) was imaged before and after extensive acceptor photobleaching by strong illumination at 561 nm in epi-fluroescence modality. (TIF) [file pone.0116900.s002.tif]

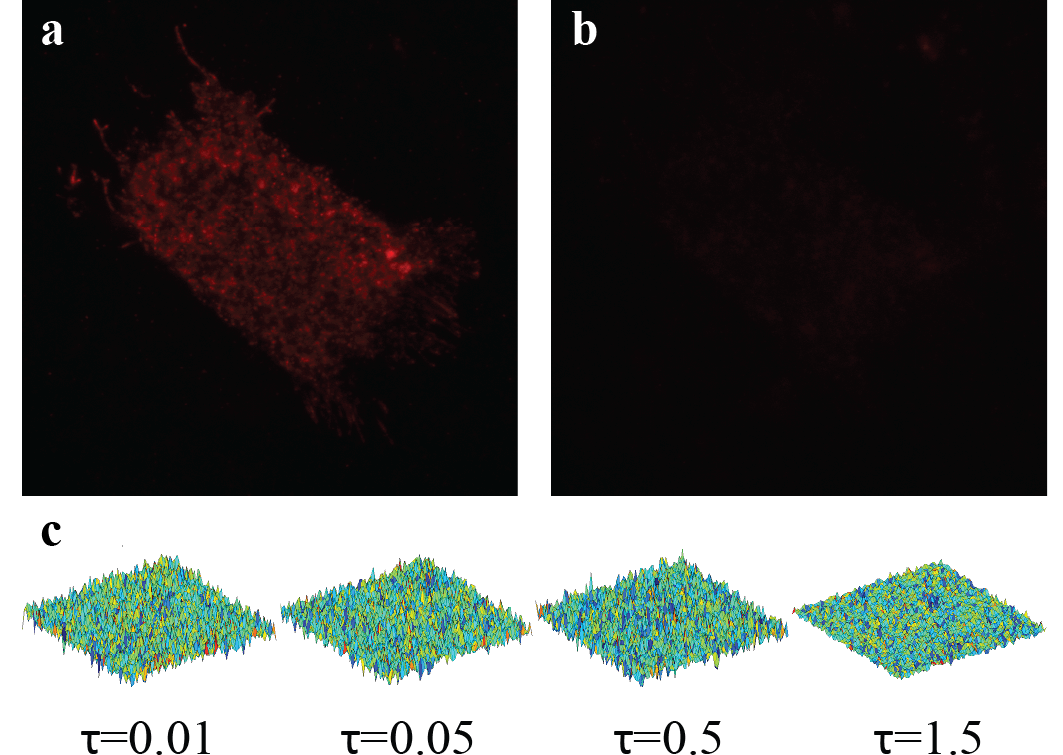

Supplement: S3 Fig — (a) TRPV1-RFP image obtained by exciting at 561 nm and acquiring between 604–620 and 650–679 nm (b) Image of the same cell acquired by exciting at 488 nm and acquiring between 604–620 and 650–679 nm. (c) Correlation function temporal evolution of image stack acquired in FRET mode (excitation at 488 nm and acquisition between 604–620 and 650–679 nm). (TIF) [file pone.0116900.s003.tif]

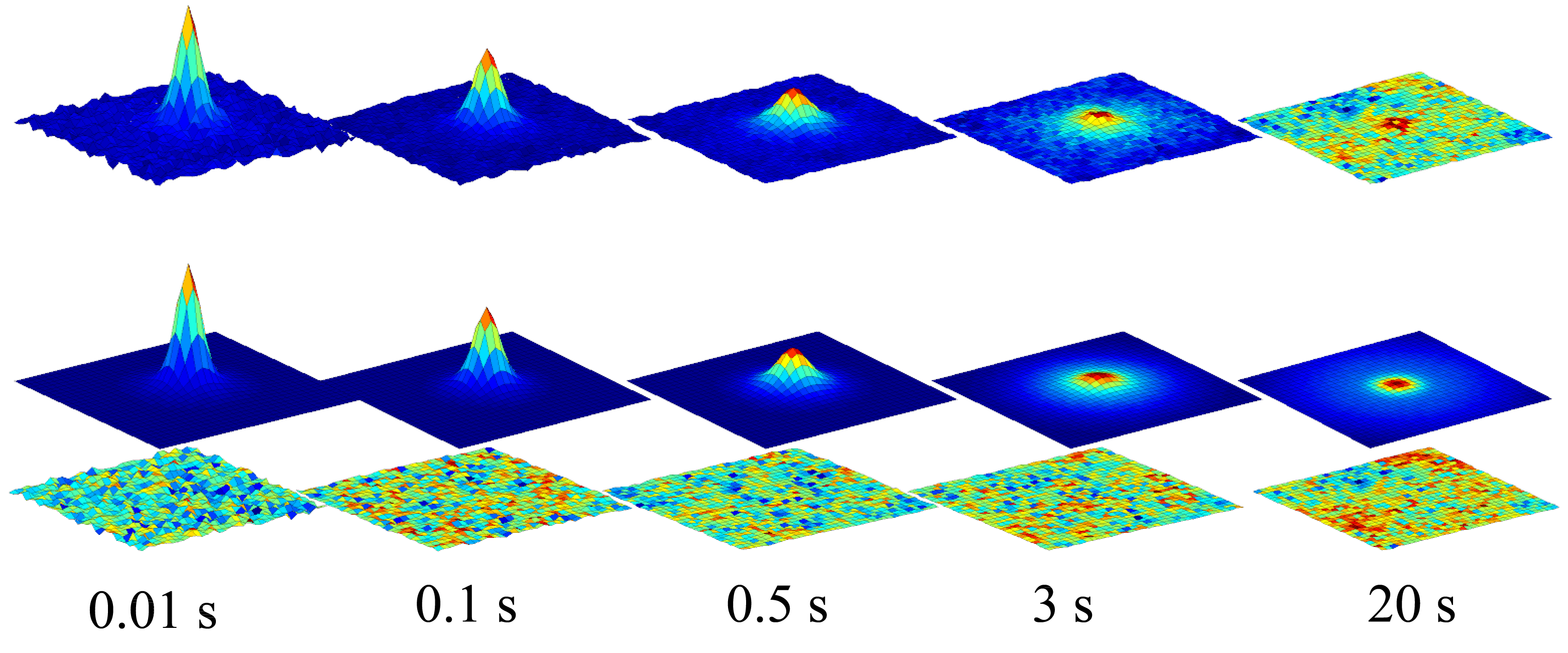

Supplement: S4 Fig — The upper row represent the correlation function, the middle the fitting and the lower row residuals. The fitting is a sum of three components that account for isotropically diffusion, binding and superdiffusion regimes. (TIF) [file pone.0116900.s004.tif]

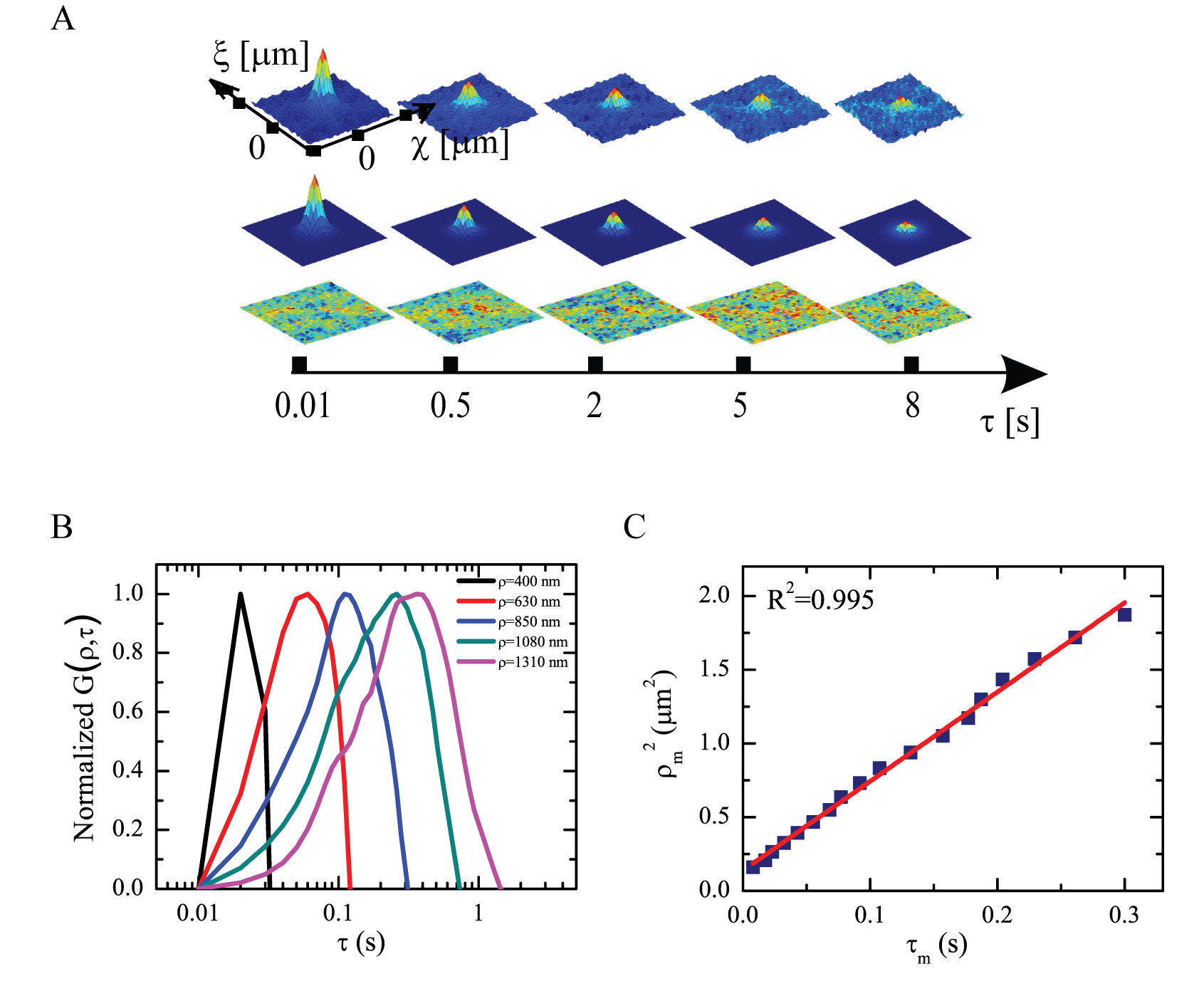

Supplement: S5 Fig — (a) Correlation function temporal evolution with the corresponding Gaussian fit and residues. The fitting is a sum of two components that account for binding and isotropic diffusion regimes. The superdiffusive TRPV1-T was not considered for the absence of intact microtubule. The residuals show that this model well described the situation in presence of nocodazole. (b) For the identification of the isotropic specie representative average correlation functions of TRPV1 for distances between about 500 nm e 1.5 μm were analyzed. The position of the maximum of the curves moves at increasing of distance along the time. The data are smoothed. The shift in the maximum clearly indicate spatial spreading of observed molecules. (c) Plot of position of each maximum identified by ρm2 and τm. The linear trend (R2 = 0.995) clearly indicates the free diffusion in this spatial scale of TRPV1-I pool with a different diffusion coefficient in respect to basal condition (D = 0.99±0.18 μm2/s). (TIF) [file pone.0116900.s005.tif]

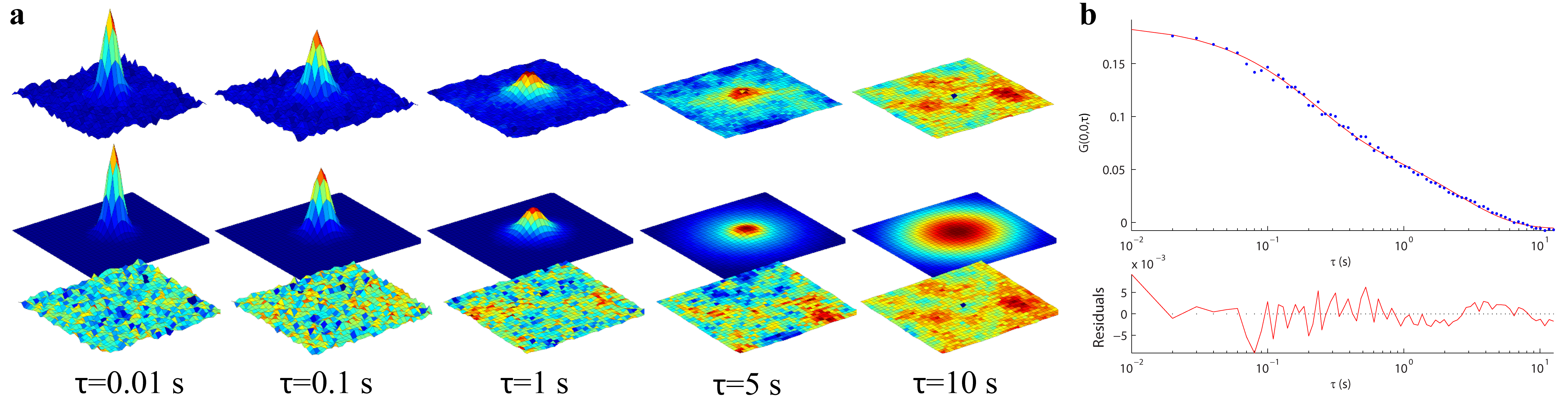

Supplement: S6 Fig — (a) Temporal evolution of the correlation function with the corresponding Gaussian fit and residues. The fitting is a sum of two components that account for binding and free diffusion regimes. (b) Temporal evolution of the components: plot of G(0,0,τ) vs. time. (TIF) [file pone.0116900.s006.tif]
