# Supplementary material for: Unveiling TRPV1 Spatio-Temporal Organization in Live Cell Membranes
Source: PLoS One. 2015 Mar 12;10(3):e0116900. doi: 10.1371/journal.pone.0116900 (PMC4357434; doi:10.1371/journal.pone.0116900)
Supplement: S1 Text — (PDF) [file pone.0116900.s007.pdf]

The mathematical and experimental details of sensitized emission FRET have been reported several times by many authors: the interested reader can find details in ref.[1-3]. Here we shall provide a short mathematical description on how the normalization procedure affects SE-FRET outcomes.

Let us define:

D = donor;
[D] = donor concentration;
A = acceptor;
[A] = acceptor concentration;
DA = D-A complex;
[DA] = D-A complex concentration;
f = [DA] / [D] ;
g = [DA] / ([D]+[DA]) ;
I_D = illumination intensity at λ_D (excitation wavelength of donor)
I_A = illumination intensity at λ_A (excitation wavelength of acceptor)
ε_D is the molar absorbance of D at λ_D, regardless of its complexation state;
ε_A is the molar absorbance of A at λ_A, regardless of its complexation state;
Φ_D = quantum yield of D, regardless of its complexation state, in the donor emission channel;
Φ_A = quantum yield of A, regardless of its complexation state, in the acceptor and FRET emission channel;
F_D = Fluorescence collected in the donor emission channel upon excitation at λ_D ;
F_A = Fluorescence collected in the acceptor/FRET emission channel upon excitation at λ_A;
F_{SE} = Fluorescence collected in the acceptor emission channel upon excitation at λ_D and due solely to energy transfer;
E = FRET efficiency;

The actual energy resonance signal (purified from donor and acceptor spectral bleed-throughs) can be expressed as:

$$F_{SE} = I_D \varepsilon_D \Phi_A E\left[DA\right] \quad \text{(S1)}$$

Let us now normalize this value by the intensity in the donor channel; we have:

$$\frac{F_{SE}}{F_D} = \frac{I_D \varepsilon_D \Phi_A E[DA]}{I_D \varepsilon_D \Phi_D \{[D] + (1-E)[DA]\}} = \frac{\Phi_A}{\Phi_D} \cdot \frac{f \cdot E}{\{1 + (1-E)f\}} \tag{S2}$$

If we now multiply both sides by the Donor to Acceptor quantum yield ratio (as suggested in ref. [4]), we obtain the apparent FRET efficiency E_D, a parameter that depends solely from E and the stoichiometric ratio f.

$$E_D = \frac{F_{SE}}{F_D} \cdot \frac{\Phi_D}{\Phi_A} = \frac{f \cdot E}{\{1 + (1-E)f\}} \tag{S3}$$

An alternative way to normalize the SE FRET signal is by the acceptor fluorescence F_A. In such a case, we have:

$$\frac{F_{SE}}{F_A} = \frac{I_D \varepsilon_D \Phi_A E[DA]}{I_A \varepsilon_A \Phi_A \{[A] + [DA]\}} = \frac{I_D}{I_A} \cdot \frac{\varepsilon_D}{\varepsilon_A} \cdot g \cdot E \tag{S4}$$

If we now multiply both sides by the acceptor to donor extinction ratio, we obtain the apparent FRET efficiency E_A:

$$E_A = \frac{F_{SE}}{F_A} \cdot \frac{\varepsilon_A}{\varepsilon_D} = \frac{I_D}{I_A} \cdot g \cdot E \tag{S5}$$

Comparison of eq. S3 and S5 shows that E_A is dependent upon the donor to acceptor excitation intensity ratio. Accordingly, for our SE-FRET analysis we adopted the donor normalization of eq. S3 to skip the apparent FRETdependence from illumination intensities.

References

1. Xia Z, Liu Y (2001) Reliable and global measurement of fluorescence resonance energy transfer using fluorescence microscopes. Biophys J 81: 2395-2402.
2. Wlodarczyk J, Woehler A, Kobe F, Ponimaskin E, Zeug A, et al. (2008) Analysis of FRET signals in the presence of free donors and acceptors. Biophys J 94: 986-1000.

3. Feige JN, Sage D, Wahli W, Desvergne B, Gelman L (2005) PixFRET, an ImageJ plug-in for FRET calculation that can accommodate variations in spectral bleed-throughs. Microsc Res Tech 68: 51-58.
4. Storti B, Bizzarri R, Cardarelli F, Beltram F (2012) Intact Microtubules Preserve Transient Receptor Potential Vanilloid 1 (TRPV1) Functionality through Receptor Binding. J Biol Chem 287: 7803-7811.
