# Supplementary material for: Unveiling TRPV1 Spatio-Temporal Organization in Live Cell Membranes
Source: PLoS One. 2015 Mar 12;10(3):e0116900. doi: 10.1371/journal.pone.0116900 (PMC4357434; doi:10.1371/journal.pone.0116900)
Supplement: S2 Text — (PDF) [file pone.0116900.s008.pdf]

Generalities and pure diffusion case. The general *i*MSD approach to STICS was already presented elsewhere [1]. Shortly, a temporal stack of images can be used to calculate the image spatiotemporal autocorrelation function $G(\xi,\chi,\tau)$, according to:

$$G(\xi,\chi,\tau)=\frac{\langle I(x,y,t)\cdot I(x+\xi,y+\chi,t+\tau)\rangle}{\langle I(x,y,t)\rangle\langle I(x,y,t+\tau)\rangle}-1, \tag{S6}$$

where $I(x,y,t)$ stands for the fluorescence intensity of the pixel located in the (x,y) position of the image at time t, and ξ, χ, τ represent variable spatial and temporal increments. Following this approach [1], the correlation function for a purely diffusing particle has the following form:

$$G_D(\rho,\tau)=\frac{g_0}{\pi\sigma_r^2(\tau)}\cdot\exp\left(-\frac{\rho^2}{\sigma_r^2(\tau)}\right), \tag{S7a}$$

where:

$$\rho^2=\xi^2+\chi^2$$

and

$$i\mathrm{MSD}(\tau)=\sigma_r^2(\tau)=4D\tau^{\alpha}+\sigma_0^2. \tag{S7b}$$

In our approach $\sigma_r^{\ 2}$ is denoted as *i*MSD since it is conceptually analogous to the mean square displacement of single particle tracking analysis. In eq. S7b D is the diffusion coefficient, α takes into account the nature of the diffusive process (α =1: isotropic diffusion, α <1 confined diffusion, α >1 guided diffusion), g_0 is a scaling factor that depends on some intrinsic properties of the fluctuations, as well as the average number of particles in the observation volume, and the *i*MSD limit for $t\rightarrow 0$ parameter σ_0 is related to the size of diffusing moiety [1]. Indeed, if σ_{xy} is the spatial resolution of the microscope on the focal plane, the diameter d of a diffusing particle can be approximated by:

$$d=\sqrt{\sigma_0^2-\sigma_{xy}^2}=\sqrt{i\mathrm{MSD}(t\rightarrow 0)-\sigma_{xy}^2}, \tag{S7c}$$

Conventionally, eq. S7a-b can be used to fit experimental data in order to recover the diffusion parameters. Yet a mathematical transformation of eq. S7a-b affords an alternative (and often simpler) way to analyze diffusion data. If we take the time derivative of $G_D(\rho,\tau)$, we obtain:

$$\frac{\partial G_D(\rho,\tau)}{\partial\tau}=\frac{g_0}{\pi}\cdot\frac{\exp\left(-\frac{\rho^2}{\sigma_r^{\ 2}(\tau)}\right)}{\sigma_r^4(\tau)}\cdot\left(\frac{\rho^2}{\sigma_r^{\ 2}(\tau)}-1\right)\cdot\frac{\partial\sigma_r^{\ 2}(\tau)}{\partial\tau}, \tag{S8}$$

Inspection of eq. S8 indicates that the plot of $G_D(\rho,\tau)$ *vs.* τ at constant ρ is characterized by a maximum where the time derivative is null and the following relation holds:

$$\left(\frac{\rho^2}{\sigma_r^{\ 2}(\tau)}-1\right)=0, \qquad \text{(S9)}$$

With help of S7b, eq. S8 with $\rho^2 > \sigma_0^2$ can be written as:

$$\rho^2 = 4D\tau_{\max}^{\alpha} + \sigma_0^2, \qquad \text{(S10)}$$

where $\tau_{\max}^{\alpha}$ stands for the time at which the $G_D(\rho,\tau)$ maximum is observed for a given ρ. Eq. S10 shows that we can recover all the relevant diffusion parameters directly from the plot of ρ^2 *vs.* $\tau_{\max}^{\alpha}$. We should note that isotropic diffusion yields a linear dependence of ρ^2 from $\tau_{\max}^{\alpha}$, whereas super and sub-diffusion processes are characterized by a non-linear plot. It is also worth to note that the plot of ρ^2 as a function of $\tau_{\max}^{\alpha}$ coincides formally with *i*MSD of the diffusive component as described in [1].

Pure binding case. The same theoretical background adopted to recover the diffusion iMSD affords also the correlation function for a pure binding process between two species not involving any diffusional motion ($G_B(\rho,\tau)$):

$$G_B(\rho,\tau)=\frac{g_0}{\pi\sigma_0^2}\cdot\exp\left(-\frac{\rho^2}{\sigma_0^{\ 2}}\right)\exp\left(-\frac{\tau}{\tau_B}\right), \qquad \text{(S11)}$$

where τ_B is the characteristic time of binding. It is worth noting that in equation S8 the time dependence is given solely by the decaying exponential containing τ_B, as the diffusion motion is assumed to be absent. Yet, eq. S7c can still be applied to recover the dimension of the spatial region where binding takes place. We should note that in this case σ_0^2 represents fully the calculated *i*MSD of the system, i.e.:

$$d=\sqrt{\sigma_0^2-\sigma_{xy}^2}=\sqrt{i\mathrm{MSD}-\sigma_{xy}^2}, \qquad \text{(S12)}$$

Mixed binding and diffusion. For an heterogeneous biological context where diffusion and binding are intertwined, a closed analytical form of $G(\rho,\tau)$ is not available. The system can be nonetheless approximated by a sum of individual components, such as:

$$G(\rho,\tau)=\sum_i A_i G_i(\rho,\tau) \qquad \text{(S13)}$$

Where $G_i(\rho,\tau)$ represent the correlation function, and A_i is the corresponding amplitude, of the *i*th component. Many factors contribute to determine the absolute amplitude of the single components and their full identification has not been accomplished yet for an arbitrary mixture of different kinds of motion. It is also worth noting that relation between the correlation amplitude and the molecular abundance strictly depends on the kind of dynamics in play. For instance, in the case of a free diffusing component, the correlation amplitude is inversely proportional to the number of molecules. Conversely, in the case of binding the "contrast" of the fluctuation increases as the affinity/binding-time increases, due to the increase of the concentration ratio between the binding site and the background. At the same time, the correlation amplitude decreases as the number of active binding points increases. Along this reasoning, the interpretation of A_i as absolute or relative abundance of a given component should be carefully avoided. Yet, A_i changes in different experimental conditions may provide some clues on how the concentration of *i* component is modulated biochemically.

In some cases fitting of the global correlation function to a sum of components can be a demanding issue. Yet we should note that for $\rho^2 \gg \sigma_0^2$ any binding contribution to $G(\rho,\tau)$ becomes negligible as compared to the diffusing components. In fact, each $G_B(\rho,\tau)$ describes a Gaussian distribution (eq. S6) whose waist does not increase with time as it does for any $G_D(\rho,\tau)$ (eq. S7a). Furthermore, with the exception of large molecular complexes, the size *d* of any particle under observation is always much smaller than σ_{xy}, and we can therefore assume $\sigma_0 \approx \sigma_{xy}$. From these considerations, we can consider $G(\rho,\tau)$ as composed only by diffusing components for $\rho^2 \gg \sigma_{xy}^2$, thereby simplifying considerably the initial problem.

FRET-iMSD. The FRET signal due to a molecular complex depends directly on the reversible formation/dissociation of the complex itself. This in turn reflects into a temporal decay of the probability to find the particles associated to the lifetime (τ_B) of FRET complex. So, in the case of *i*MSD evaluated in FRET modality (i.e. *i*MSD calculated on the FRET signal and pertaining solely to the complex) we can define a complete correlation function that accounts for both diffusion and binding time of the complex as:

$$G_{FRET}(\rho,\tau) = \frac{g_0}{\pi\sigma_r^2(\tau)} \cdot \exp\left(-\frac{\rho^2}{\sigma_r^2(\tau)}\right)\exp\left(-\frac{\tau}{\tau_B}\right), \qquad \text{(S14)}$$

References

1. Di Rienzo C, Gratton E, Beltram F, Cardarelli F (2013) Fast spatiotemporal correlation spectroscopy to determine protein lateral diffusion laws in live cell membranes. Proc Natl Acad Sci U S A.
2. Digman MA, Gratton E (2009) Analysis of diffusion and binding in cells using the RICS approach. Microsc Res Tech 72: 323-332.
